# Function Annotation of an SBP-box Gene in *Arabidopsis* Based on Analysis of Co-expression Networks and Promoters

**DOI:** 10.3390/ijms10010116

**Published:** 2009-01-02

**Authors:** Yi Wang, Zongli Hu, Yuxin Yang, Xuqing Chen, Guoping Chen

**Affiliations:** 1 College of Bioengineering, Chongqing University, Chongqing 400030, P.R. China. E-Mails: dna.wangyi@yahoo.com.cn (Y. W.); huzongli71@yahoo.com.cn (Z. H.); yangyuxin@sina.com.cn (Y. Y.); chenxvqing@sina.com (X. C.); 2 Beijing Research Center of Agro-Biotechnology, Beijing 100089, P.R.China

**Keywords:** Arabidopsis, SPL, co-expression, network, promoter

## Abstract

The SQUAMOSA PROMOTER BINDING PROTEIN–LIKE (SPL) gene family is an SBP-box transcription family in *Arabidopsis*. While several physiological responses to SPL genes have been reported, their biological role remains elusive. Here, we use a combined analysis of expression correlation, the interactome, and promoter content to infer the biological role of the SPL genes in *Arabidopsis thaliana*. Analysis of the SPL-correlated gene network reveals multiple functions for SPL genes. Network analysis shows that SPL genes function by controlling other transcription factor families and have relatives with membrane protein transport activity. The interactome analysis of the correlation genes suggests that SPL genes also take part in metabolism of glucose, inorganic salts, and ATP production. Furthermore, the promoters of the correlated genes contain a core binding cis-element (GTAC). All of these analyses suggest that SPL genes have varied functions in *Arabidopsis*.

## 1. Introduction

Transcription factors (TFs) are DNA-binding proteins that regulate gene expression at the level of mRNA transcription. In plants, as in all living organisms, transcription factors are an important level of gene regulation. Many families of transcription factors have been identified in plants. Analysis of the *Arabidopsis* genome reveals 29 classes of transcription factors, 16 of which appear to be unique to plants [1]. One of these contains a DNA binding domain referred to as the SQUAMOSA PROMOTER BINDING PROTEIN (SBP) domain and is encoded by the SBP-box, a feature characteristic of the *Arabidopsis* SQUAMOSA PROMOTER BINDING PROTEIN–LIKE (SPL) gene family [2, 3].

The first SBP-domain proteins, which were isolated from snapdragon [4], showed *in vitro* binding to a sequence motif in the promoter region of the floral meristem identity gene SQUAMOSA [5]. Since then, SBP-box genes have been identified in many plants. For example, the *Arabidopsis* genome alone contains 16 SPL genes [3].

A number of physiological and biochemical studies have implicated the SBP-box gene in the regulation of plant tissue development. SPL3 may interact *in vivo* with promoters of the AP1 or CAL MADS-box genes of the SQUAMOSA family to act as a positive transcriptional regulator modulating floral development [2]. An SPL gene is involved in the early stages of microsporogenesis and megasporogenesis [6] and as well as the development of normal plant architecture [7]. This family of genes was also associated with maize kernel development [8] and tomato fruit ripeness [9]. Very recently, a study showed that the SBP-box genes SPL9 and SPL15, which are regulated by microRNAs, control shoot maturation in *Arabidopsis* [10].

Despite an increasing body of physiological and biochemical data, the biological role of the SBP-box transcription family remains elusive. In order to infer a biological role for it, we took advantage of the large repositories of *Arabidopsis thaliana* microarray data to study the co-expression gene with SPL. We identified 112 genes whose expression most highly correlated with them in response to various treatments and during development. Furthermore, we analyzed the promoters of these genes in order to identify the regulatory motifs. The results of our study predict that the SBP-box transcription family is involved in plant tissue development, responses to abiotic and biotic stresses, and the activation of other transcription factors and membrane proteins. Furthermore, we demonstrate how computational analyses linking expression data with regulatory information from studies of promoter elements can provide novel insights into the function of a transcription family whose function is unclear.

## 2. Materials and Methods

### 2.1. Identification of correlated genes and GO analysis

A total of 1,779 Affymetrix raw data Cel files were downloaded from the AtGenExpress and GEO sites [11–13]. All of them were derived from the Affymetrix ATH1 gene GeneChip microarray for Arabidopsis, and the samples contained at least two replicate chips. The required probe set-to-locus mappings for the ATH chip were obtained from TAIR (ftp://ftp.arabidopsis.org/home/tair/Microarrays/Affymetrix, version 2-5-2007).

Normalization of the raw data Cel files was performed in R using the MAS5 algorithms, which are implemented in the affy package available from the BioConductor project [14, 15]. R scripts were used to calculate non-parametric correlation coefficients (Spearman’s rho) between the expression of SPL genes and the expression of each of the ~22,000 genes represented on the Affymetrix array used to generate this dataset. We ranked the genes according to the correlation coefficients and reported genes that were most positively correlated with SPL gene expression. The p-values were calculated using bivariate normal distribution, with p representing the probability of observing an equal or larger positive or negative correlation by chance. For details of the method, see the article by Wei [16].

To characterize the correlated genes, the web-based ‘Classification SuperViewer’ program (http://bar.utoronto.ca/ntools/cgi-bin/ntools_classification_superviewer.cgi) was used to search for differential distributions of gene ontology (GO) and biological terms within the correlation genes.

### 2.2. Arabidopsis interactome network and clustering analysis

The *Arabidopsis* interactome network was built by the AtPID. The AtPID (*Arabidopsis thaliana* Protein Interactome Database) represents a centralized platform for depicting and integrating information pertaining to protein-protein interaction networks, domain architecture, ortholog information, and GO annotation in the *Arabidopsis thaliana* proteome. We mapped the correlated genes to the network and also selected the first neighbors of the genes. To identify the function of SPL-correlated proteins in network clusters, the latest versions of the Biological Network Gene Ontology (BiNGO) [17] and GOlorize tools [18] were used for the statistical evaluation of groups of proteins. This evaluation was carried out using existing annotations of the Gene Ontology Consortium (http://www.geneontology.org).

### 2.3. Promoter analysis

Applications in the web-based promoter analysis program Promomer [19] were used to analyze the promoters (−1 kb upstream of the predicted translation start site) of the 112 genes showing the strongest correlations to SPL genes. The Promomer program aims to provide a user-friendly, web-based interface to accomplish two goals: (1) to identify statistically over-represented elements in a gene or a group of genes in *Arabidopsis* using the enumerative method, and (2) to find the number and position of occurrences of an element in genes across the *Arabidopsis* genome or in a subgroup of genes.

## 3. Results

### 3.1. Expression correlation and gene ontology (GO) analyses

**Figure 1.**
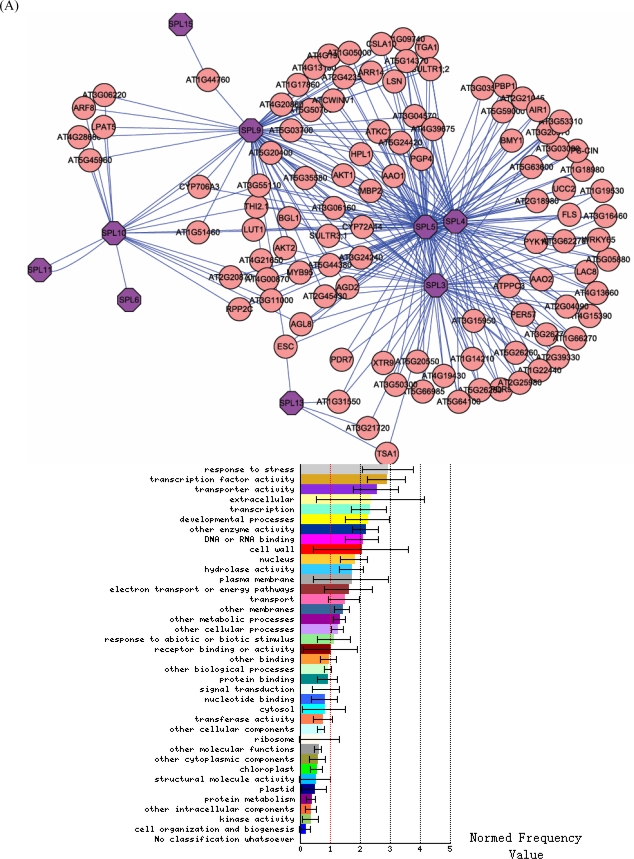

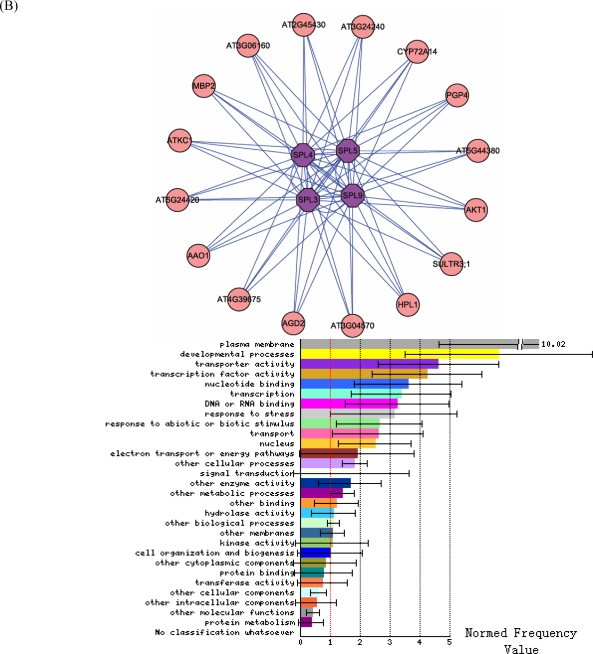
Co-expression network and GO analysis of SPL genes. (A) A network composed of SPL and correlated genes. GO analysis shows that the network is involved in extensive functions. (B) A complex within the co-expression network and its function.

In the first step of the analyses, we extracted and ranked the 112 genes whose expression most tightly correlates with that of SPL genes (Table 1). We took advantage of a visualization tool called Cytoscape to view expression data and analysis results. Using the available data regarding correlations between different *Arabidopsis* genes, we constructed a network composed of the SPL genes and their co-expressed genes. This network contains 112 *Arabidopsis* genes (nodes) and 294 correlations (edges) (Figure 1A). Three interesting facts are observed from the co-expression network. First, some SPL genes are absent from the network, including SPL1, SPL2, SPL6, SPL7, SPL8, SPL12, SPL14 and SPL16. This reflects the current lack of knowledge regarding correlations between these SPL genes and other genes. Second, many genes correlate with SPL3, SPL4, SPL5, and SPL9; some genes correlate with SPL10 and SPL13; while SPL11 and SPL15 have only one co-expression gene. Third, within the co-expression network, there is a region comprising four SPL genes that contain 20 *Arabidopsis* genes (nodes) and 79 correlations (edges) (Figure 1B).

In order to identify a functional role for SPL genes, the correlated genes were analyzed in a web-based gene ontology (GO) analysis tool to identify any bias in GO functional annotation terms in the correlated genes compared to the remainder of the *A. thaliana* genome (Supplementary Table 1). When GO clustering was applied to the 112 co-expression genes, some GO biological categories were retrieved. Most of these related to stress response, transporter activity, and transcription factor activity (Figure 1A). Other biological processes identified were associated with extracellular activities, development processes, and enzyme activity (Figure 1A). Thus, given previous work [3] suggesting that SPL genes play a role in controlling plant development, we can infer that SPL genes may not only do this but also take part in many other biological processes. In the co-expression network, a complex related to development and the plasma membrane was found (Figure 1B). We presume that the complex is very important for SPL gene function of development (Figure 1B), and SPL proteins may form heterodimers or multimers to carry out their functions.

### 3.2. Interactome analysis

**Figure 2.**
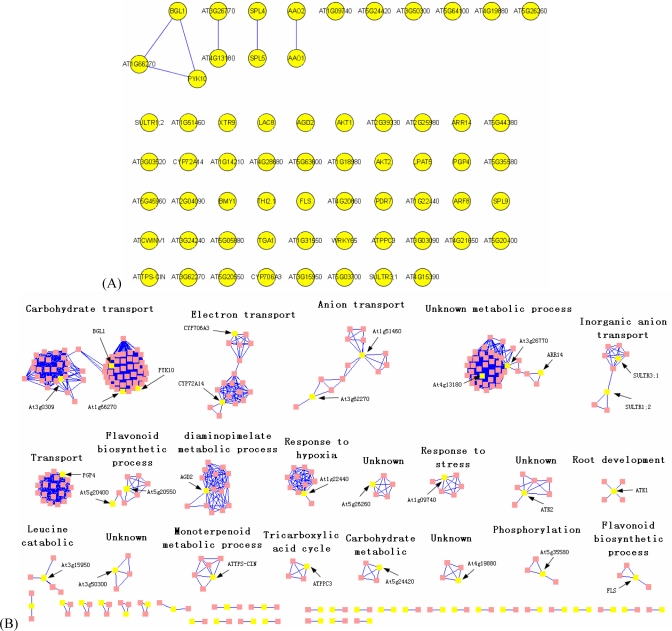
Network analysis of the *Arabidopsis* interactome showing direct interactions between co-expressed gene products and their first neighbors. (A) Direct interactions between correlated gene products. (B) Direct interactions between correlated gene products and their first neighbors. Some sub-networks show enrichment in GO terms (functional modules). Arrows indicate the SPL-correlated gene nodes present in these functional modules.

To evaluate functional associations between co-expressed proteins, we mapped the genes whose expression was correlated with SPL genes using the *Arabidopsis thaliana* interactome network (Figure 2A). Since pairs of interacting proteins are more likely to contain similar GO annotations than pairs of unrelated proteins, annotations of neighbor proteins in the network can be used to make predictions of functions. In particular, the GO annotations allow functional assignment of uncharacterized gene products, such when At3g62270 was identified in a large-scale interactome mapping study of anion transport (Figure 2B).

Cluster analysis of this network suggested the molecular mechanisms underlying the function of SPL genes. Analysis of the connected sub-graphs and their GO terms identified functional modules enriched for carbohydrate transport, electron transport, anion transport, flavonoid biosynthetic processes, diaminopimelate metabolic processes, response to hypoxia and stress, and root development (Figure 2B). Many genes and proteins co-expressed with SPL genes are located in these modules, which supports their role in a diversity of physiology processes. These observations support the theory that the network modeled here constitutes a framework that can guide in-depth experimental study of genes and proteins related to SPL gene function in *Arabidopsis*.

### 3.3. Promoter analysis

The shared expression profiles of the 112 correlated genes in response to development and stress suggests that these genes are under the same regulatory control and are thus likely to share cis-elements in their promoter regions. Since these genes are co-expressed with SPL genes, we hypothesized that they might be directly regulated by SPL genes. The cis-element of the SBP-box transcription factor is unclear. To reveal which DNA motif can bind to SPL genes, we analyzed the promoter regions of these genes 1 kb upstream of the predicted transcription start site. We also analyzed aspects of shared transcriptional activation sites on these genes for the presence of known plant cis-elements.

**Figure 3.**
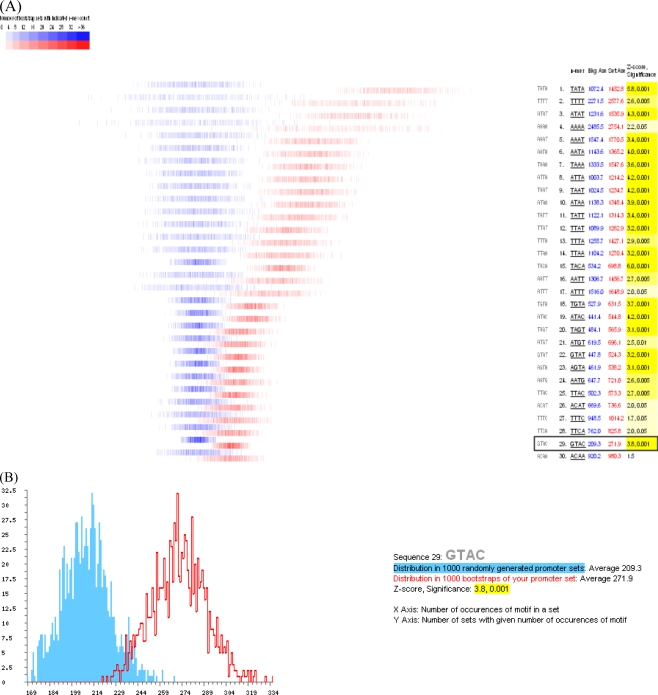
Promoter analysis of SPL co-expression genes. (A) The distribution of the occurrence of the GTAC motif in 1000 sets of SPL-correlated genes promoters randomly selected from the *Arabidopsis* genome. (B) Distribution in 1000 randomly generated promoter sets: Average 209.3. Distribution in 1000 bootstraps of our promoter set: Average 271.9. Z-score, Significance: 3.8,0.01.

Based on previous research [3, 20–22], we hypothesized that the GTAC motif was the cis-element common to the SBP-box genes. Indeed, our analysis showed the GTAC motif to be a statistically over-represented element (Figure 3). Two distributions were created from 1,000 bootstrapped sets, each containing the same number of genes. The first was obtained by sampling for the frequency of occurrence of an element from the correlated genes cluster promoter set bootstrapped 1,000 times; the second was obtained from the same bootstrapping process carried out on 1,000 whole genome promoter data sets containing the same number of sequences as the cluster set. The distribution of occurrence of a given element in both data sets was then obtained and plotted [23] and significant differences were highlighted (Supplementary Figure 1). For comparison, significant tetramers whose expression is unrelated to that of SPL genes are also shown.

The analysis (Supplementary Table 2) indicates that the invariant core GTAC motif is present in 100/112 of our correlated genes a total of 271.9 times, which is higher than the average of 209.3 times across 1,000 randomly-generated promoter sets (Figure 3). The promoter analysis indicates that multiple copies of the SBP-box elements are present in a high percentage of SPL-correlated genes. The core GTAC motifs are significantly enriched compared to expected frequencies, suggesting that they are important regulatory elements in these co-expressed genes.

## 4. Discussion

Although transcription factors are generally low expressed, microarray has been widely used for transcription factors expression research [24–26]. With recent interest in co-expression, transcription factors co-expression has emerged as a novel holistic approach for their function analysis. For example, MADS-box proteins form specific homo- and heterodimers and even higher order complexes to conduct their functions [27, 28]. Specific interactions among MADS proteins require that they are present in the same cells and tissues under the same developmental stages, and correspondingly it has been shown that transcripts with overlapping expression patterns are preferred as protein interaction partners [29, 30]. Roosa *et al*. used co-expression method identify correlation genes with MADS in *Gerbera hybrida*, they found that MADS proteins not only might correlate with themselves but also correlated with MYB factors [31]. In our analysis, we used microarray data to classify co-expression network of SPL genes. We think the network may help us to infer the function of SPL genes.

### 4.1. SPL genes may play a key role in the stress response

Transcriptional control of the expression of stress-responsive genes is a crucial part of plant responses to a range of abiotic and biotic stresses. Research carried out in recent years [23] has been productive in identifying transcription factors that are important for regulating plant responses to these stresses. SPL genes have not been shown to be affected by stress in *Arabidopsis*. Nevertheless, our GO analysis revealed that some genes correlated with SPL genes were involved in the stress response (Supplementary Table 1). These genes include P57 (GO: 0006979, response to oxidative stress), AKT1 (GO: 0009651, response to salt stress), At3g16460 (GO: 0009409, response to cold), ATPPC3 (GO: 0009414, response to water deprivation), and ATCWINV1 (GO: 0009611, response to wounding).

In addition, two transcription factors (TGA1 and WRKY65) that belong to the bZIP and WRKY families, respectively, are co-expressed with SPL genes. bZIPs are a large family of transcription factors in plants, and 75 members are present in *Arabidopsis* [32]. One class of bZIP proteins that is linked to stress responses comprises the TGA/octopine synthase (ocs)-element-binding factor (OBF) proteins. These bind to the activation sequence-1 (as-1)/ocs element, which regulates the expression of some stress-responsive genes such as PR-1 and GLUTATHIONE S-TRANSFERASE6 (GST6) [33, 34]. In *Arabidopsis*, seven members of the TGA/OBF family play a role in plant defense, xenobiotic stress responses, and development. WRKY proteins are a novel family of transcription factors that are unique to plants and that form a large, 74-member family in *Arabidopsis*. WRKY proteins contain either one or two WRKY domains, which is 60-residue region that contains the amino acid sequence WRKYGQK and a motif similar to zinc fingers. Certain WRKY family members show enhanced expression or DNA-binding activity following induction by a range of pathogens, defense signals, and wounding [35]. Since the two transcription families are very important for plant response to stress, and because SPL genes can control some stress-responsive genes, we speculate that SPL genes also play a key role in the stress response.

### 4.2. A complex control network linking SPL genes and other transcription families

Our GO analysis shows transcription activity to be an important function for co-expressed genes. SPL genes can activate other transcription factor families, including B3, bZIP, WRKY, MYB, bHLH, and MADs-box. We believe that SPL genes function by controlling the expression of these transcription families. For example, the expression of AGL8 (FUL), a MADs-box gene, correlates with SPL gene expression. In another study, the MADs-box gene AP1 was also found to be activated by SBP-box genes [2]. The proteins AP1 and FUL may serve as hubs between the flower induction pathway, which is comprised of interacting proteins such as SVP, SOC1, and AGL24, and the floral organ identity proteins. Both AP1 and FUL have dual functions in floral meristem identity (early function) and floral organ determination (late function) [36, 37]. This is consistent with the fact that the flowering proteins and floral homeotic proteins come together to form dimers.

**Figure 4.**
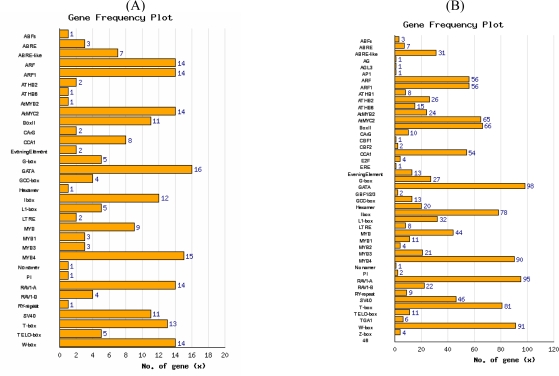
Known *cis-*element analysis of (A) SPL genes and (B) genes co-expressed with SPL genes.

Analysis of cis-elements suggests an even more complex picture: (1) SPL genes may be activated by other transcription families (Figure 4A), and (2) the 112 genes correlated to SPL genes in our analysis may be controlled by other transcription families (Fig 4B). We therefore hypothesize a complex control network linking SPL genes with other transcription families.

### 4.3. SPL genes and integral membrane proteins

An integral membrane protein (IMP) is a protein molecule (or assembly of proteins) that is permanently attached to the biological membrane. In the co-expression network of SPL genes, some IMP genes correlate with SPL genes. These include ATK1 and ATK2 (ARABIDOPSIS K TRANSPORTER), ATVGT1 (sugar transporter family protein), PGP4 (P-GLYCOPROTEIN 4), SULTR1;2 (SULFATE TRANSPORTER 1;2), At3g55110 (ABC transporter family protein), and some genes encoding ligands of membrane proteins. The IMPs encoded by these genes include transporters, channels, receptors, enzymes, structural membrane-anchoring domains, proteins involved in accumulation and transduction of energy and sugar, and proteins responsible for the stress response. Our analysis of genes correlated with SPL genes leads us to speculate that SPL genes may participate in development, stress responses, and other biological processes by controlling the expression of IMPs. In addition, some SPL proteins have a transmembrane domain, which suggest that SPL may have interaction with IMPs.

### 4.4. SPL genes may regulate themselves

**Figure 5.**
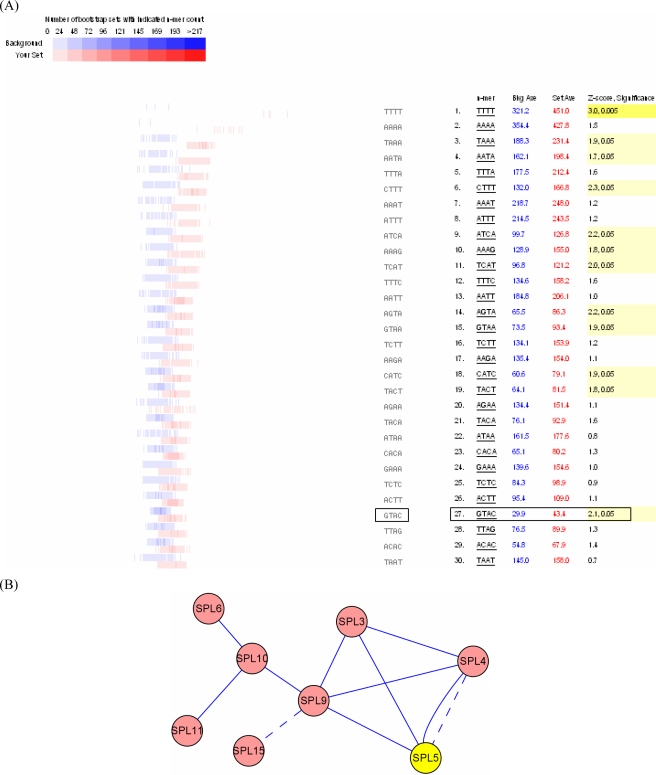
Promoter and network analysis of SPL genes. (A) The distribution of the occurrence of the GTAC motif in 1000 sets of SPL genes promoters randomly selected from the *Arabidopsis* genome. Distribution in 1,000 randomly generated promoter sets: Average 29.7. Distribution in 1,000 bootstraps of our promoter set: Average 42.9. Z-score, Significance: 2.2,0.05. (B) A putative network of SPL genes (a solid line indicates co-expression, and a dashed line indicates protein interactions).

Our analysis indicates that the cis-element shared by SPL genes, the GTAC motif, is necessary for binding of SPL proteins to DNA. In the co-expression network, the expression of some SPL genes correlates with that of other SPL genes; these genes may form a complex within the network (Figure 1B). Therefore, we propose that SPL genes regulate themselves. Using promoter analysis, we found the GTAC motif to be present in the 1 kb upstream region in 13/16 of SPL genes a total of 56 times (Supplementary Table 3), which was significantly higher than the occurrence in tetramer sets of randomly selected promoters (Figure 5A). In the AtPID database [38], we found that SPL9 can interact with SPL15, and SPL4 can interact with SPL5. SPL genes may therefore regulate themselves using a feedback loop based on protein interactions and transcriptional regulation (Figure 5B).

### 4.5. SPL genes have varied functions

The SBP-box gene family belongs to a group of plant-specific zinc finger protein genes that encodes plant-specific transcription factors. The proteins encoded by SBP-box genes bind specifically to promoters of the floral meristem identity gene SQUAMOSA and its orthologous genes [2, 3, 6]. Except for the presence of a conserved SBP-box/domain, the SPL genes vary substantially in their genomic organization, transcript size, and the size and sequences of their encoded proteins. The position of the SBP domain within the protein also varies [39]. The different gene structures and the divergence of amino acid sequences among different SPL genes provide us with some hints that SBP transcription factors may have a variety of physiological functions. To date, several important and divergent biological processes regulated by SBP-box genes have been reported. These include flower and fruit development [2, 4, 8, 9], architecture formation [40], sporogenesis [6], response to copper and fungal toxin [7, 41], and control of GA level [42].

Our co-expression analysis has revealed that SPL genes may play a role in the stress response. Network analysis suggests that SPL genes function by controlling other transcription factor families, and that they have relatives encoding proteins involved in membrane protein transport. The interactome of the correlated genes has shown that SPL genes may also take part in glucose, inorganic salt, and ATP production. Notably, some SPL genes are not detected in our co-expression analysis. Among them, SPL8 can affect pollen sac development [6], and SPL14 plays a role in plant development and sensitivity to fumonisin B1 [7]. All of the available evidence suggests that SPL genes have varied functions in *Arabidopsis*.

## 5. Conclusion

The SPL gene family belongs to a group of plant-specific zinc finger protein genes that encodes plant-specific transcription factors. The expression of SPL genes are significantly correlated with that of genes involved in the defense response pathway in response to various biotic and abiotic stress. The expression of SPL genes are correlated with other transcription factors gene and some integral membrane proteins. Additionally, like the MADS-box genes, some SPL genes are correlated with themselves. Furthermore, the promoters of the correlated genes contain a core binding cis-element (GTAC). All of these analyses suggest that SPL genes have varied functions in *Arabidopsis*.

## Appendix: Supplementary Material

Supplementary material is available from http://dx.doi.org/10.3390/ijms10010116

## Figures and Tables

**Table 1.** List of genes that are expression correlated with SPL in our analysis.

AGI ID	Annotation	AGI ID	Annotation
AT5G20960	ALDEHYDE OXIDASE 1	AT3G18850	LPAT5__LPAT5
AT1G52030	MYROSINASE-BINDING PROTEIN 2	AT4G00870	basic helix-loop-helix (bHLH) family protein
AT2G26650	ARABIDOPSIS K TRANSPORTER 1	AT5G37020	ARF8
AT3G55110	ABC transporter family protein	AT3G09260	phosphate starvation-response 3.1 oxidoreductase, 2OG-Fe(II) oxygenase family
AT4G15440	HYDROPEROXIDE LYASE 1	AT5G20400	protein
AT3G06160	transcriptional factor B3 family protein	AT4G19430	unknown protein
AT5G44380	FAD-binding domain-containing protein	AT5G17820	peroxidase 57 (PER57) (P57) (PRXR10)
AT5G60910	FUL	AT3G06220	DNA binding / transcription factor
AT2G42200	SPL9	AT5G45960	GDSL-motif lipase/hydrolase family protein
AT2G45430	DNA-binding protein-related	AT4G21650	subtilase family protein UCC2__UCC2 (UCLACYANIN 2); copper
AT3G04570	DNA-binding protein-related	AT2G44790	ion binding
AT4G32650	ARABIDOPSIS THALIANA K+ RECTIFYING CHANNEL 1	AT3G28500	60S acidic ribosomal protein P2 (RPP2C)
AT3G14680	CYP72A14	AT4G12550	Auxin-Induced in Root cultures 1
AT1G20900	ESCAROLA	AT4G20860	FAD-binding domain-containing protein
AT1G60680	ARF-GAP DOMAIN 2	AT4G28680	tyrosine decarboxylase, putative
AT3G53130	LUTEIN DEFICIENT 1	AT5G63600	flavonol synthase, putative
AT3G51895	SULFATE TRANSPORTER 1	AT2G01760	ARABIDOPSIS RESPONSE REGULATOR 14
AT2G47000	P-GLYCOPROTEIN 4	AT5G59000	zinc finger (C3HC4-type RING finger) family protein
AT4G39675	unknown protein	AT5G03700	PAN domain-containing protein
AT5G24420	glucosamine/galactosamine-6-phosphate isomerase-related	AT2G21045	similar to unknown protein
AT3G24240	leucine-rich repeat transmembrane protein kinase, putative	AT5G50760	auxin-responsive family protein
AT1G72260	toxin receptor binding	AT3G15950	(TSA1-LIKE); unknown protein
AT3G15270	SPL5	AT1G17860	trypsin and protease inhibitor family protein
AT2G33810	SPL3	AT2G20870	cell wall protein precursor, putative
AT1G53160	SPL4	AT5G20550	oxidoreductase
AT5G05880	UDP-glucosyl transferase family protein	AT1G05000	tyrosine specific protein phosphatase family protein
AT1G24070	Cellulose synthase-like A10	AT3G43600	AAO2
AT3G20370	MATH domain-containing protein	AT4G22200	Arabidopsis K+ transporter 2
AT5G26280	MATH domain-containing protein	AT3G21720	isocitrate lyase, putative
AT1G51460	ABC transporter family protein	AT1G52410	TSK-ASSOCIATING PROTEIN 1
AT3G16460	jacalin lectin family protein	AT4G19880	similar to unknown protein
AT3G53480	PLEIOTROPIC DRUG RESISTANCE 9	AT2G18980	peroxidase, putative
AT5G44620	CYP706A3	AT1G18980	germin-like protein, putative
AT5G01040	laccase 8	AT4G13180	short-chain dehydrogenase
AT5G65210	TGA1	AT5G63590	Flavonol synthase
AT1G52400	BGL1	AT3G03090	ATVGT1__sugar transporter family protein
AT5G14370	similar to CIL	AT3G25820	TERPENE SYNTHASE-LIKE SEQUENCE-1,8-CINEOLE
AT1G66270	beta-glucosidase	AT4G15210	BETA-AMYLASE
AT5G02030	HB-6_RPL_LSN_BLH9_BLR_PNY_RPL_VAN__LSN	AT2G42350	zinc finger family protein
AT4G13660	pinoresinol-lariciresinol reductase, putative	AT3G50300	transferase family protein
AT4G15390	transferase family protein	AT3G03520	phosphoesterase family protein
AT5G64100	peroxidase, putative	AT1G15210	PLEIOTROPIC DRUG RESISTANCE 7
AT1G78000	SULFATE TRANSPORTER 1;2	AT3G16420	PYK10-BINDING PROTEIN 1
AT1G29280	WRKY DNA-binding protein 65	AT3G13790	ARABIDOPSIS THALIANA CELL WALL INVERTASE 1
AT2G39330	jacalin lectin family protein	AT3G62270	anion exchange family protein
AT1G09740	ethylene-responsive protein, putative	AT4G25820	XYLOGLUCAN ENDOTRANSGLYCOSYLASE 9
AT1G22440	alcohol dehydrogenase, putative	AT1G27370	SPL10
AT5G26260	MATH domain-containing protein	AT5G66985	unknown protein
AT2G25980	jacalin lectin family protein	AT3G26770	short-chain dehydrogenase
AT1G31550	carboxylic ester hydrolase/lipase	AT3G11000	similar to kelch repeat-containing protein
AT1G19530	unknown protein	AT3G14940	PHOSPHOENOLPYRUVATE CARBOXYLASE 3
AT1G74430	AtMYB95	AT1G44760	universal stress protein (USP) family protein
AT2G04090	MATE efflux family protein	AT1G14210	ribonuclease T2 family protein
AT5G35580	kinase	AT1G27360	SPL11
AT3G53310	transcriptional factor B3 family protein	AT1G69170	SPL6
AT5G50570	SPL13	AT3G57920	SPL15
